# Antitumor immunity induced by VE-cadherin modified DC vaccine

**DOI:** 10.18632/oncotarget.18654

**Published:** 2017-06-27

**Authors:** Jing Zhou, Yufeng Xi, Xiyan Mu, Rongce Zhao, Hongdou Chen, Li Zhang, Yang Wu, Qiu Li

**Affiliations:** ^1^ The Department of Medical Oncology, Cancer Center, State Key Laboratory of Biotherapy/Collaborative Innovation Center for Biotherapy, West China Hospital, Sichuan University, Sichuan, China; ^2^ State Key Laboratory of Biotherapy and Cancer Center/Collaborative Innovation Center for Biotherapy, West China Hospital, Sichuan University, Sichuan, China; ^3^ Department of Gynecology and Obstetrics, Key Laboratory of Obstetrics and Gynecologic and Pediatric Diseases and Birth Defects of Ministry of Education, West China Second Hospital, Sichuan University, Sichuan, China; ^4^ Division of Liver Transplantation, Department of Liver Surgery, West China Hospital, Sichuan University, Sichuan, China

**Keywords:** DC, VE-cadherin, vaccine, anti-angiogenesis, immunoregulation

## Abstract

Dendritic cells (DCs) are the most potent antigen-presenting cells. A strong interest has been developed in DC vaccines for cancer immunotherapy. Besides, angiogenesis is essential for tumor growth. VE-cadherin has a crucial function in various aspects of vascular biological functions. Here, we produced the full VE-cadherin gene modified DC vaccine (DC-VEC). Its antitumor immunity and chief mechanism driving antitumor effect was evaluated. Analyses were performed including test of antitumor antibody, CTL-mediated cytotoxicity experiment, vascular density, evaluation of the variation of cells and cytokines in immunoregulation. Its damage to the major organs was also evaluated. DC-VEC vaccine resulted in retarded tumor progression and prolonged survival in mice. In DC-VEC group, large amount of immunoglobulin was generated, T cells exhibited greater cytotoxicity against VE-cadherin, and tumor angiogenesis was suppressed. Besides, a decrease of VEGF-A and TGF-β1, and an increase of IL-4 and IFN-γ were observed. CD4^+^ and CD8^+^ T cells were higher, with increased IFN-γ secretion. The percentage of myeloid-derived suppressor cells and regulatory T cells decreased mildly. Also, it had no pathologic changes in major organs. DC-VEC vaccine represents a promising antitumor immunotherapy. The main mechanism is associated with its anti-angiogenesis and immunoregulation response.

## INTRODUCTION

Dendritic cells (DCs) are the most potent antigen-presenting cells and a strong interest has been developed in their use of cancer immunotherapy [1–3]. Transport of antigen by DCs is probably of key importance to initiate immune response, thus permitting the establishment of immunological memory [4, 5]. These cells can induce both the generation and proliferation of specific cytotoxic T lymphocytes (CTLs) and helper T cells via antigen presentation by MHC class I and class II molecules in different settings. Besides, DCs may directly activate the growth and differentiation of B lymphocytes. The role of DCs in humoral responses has been documented *in vitro* and vivo [6, 7].

In the past decades, more than 100 preclinical studies had analyzed DC-targeting approaches that induced T cells and antibody responses. Much attention has been paid toward the use of DCs in vaccine strategies for the treatment of cancer. In these experimental tumor models, DCs were pulsed with tumor-associated antigens in various forms, including whole tumor cells, cell lysates, peptides, proteins, RNA, DNA, or DCs fused with tumor cells [8, 9]. A requirement for all of these approaches is to acquire and present tumor-specific antigens by DCs. Recently, several reviews of clinical trials conducted with DC-based vaccines in cancer patients have demonstrated that DC vaccines could induce immunotherapy upon optimization of different parameters and is indeed a prime candidate for the treatment of various cancers [10, 11].

In addition, immunotherapy involves the use of vascular endothelial-cadherin (VE-cadherin), which is an endothelial cell-specific adhesion molecule localized at cell–cell contact regions that is regarded as adherence junctions. VE-cadherin has a crucial role in various aspects of vascular biological functions, including endothelial cell migration, survival, contact-induced growth inhibition, vascular integrity and endothelial cell assembly into tubular structures [12–15]. As an important mediator in the developmental angiogenesis, VE-cadherin is a potential target for anti-tumor therapy. Monoclonal antibodies against VE-cadherin have been shown to be able to inhibit the tumor growth and metastasis *in vitro* [12, 16]. We have demonstrated previously that mannan modified VE-cadherin is an attractive vaccine strategy for cancer immunotherapy [17].

In recent years, the anti-angiogenesis therapy has become one of the most important strategies for the treatment of cancers. There have been new targeted drugs available against tumor angiogenesis. However, due to several factors during the process of tumorigenesis, such as the existence of multiple angiogenesis related signaling pathways, immune escape by tumor antigen modulation or reduction its immunogenicity, etc., anti-angiogenesis therapy faces great challenge [18–21]. Therefore, combining DCs and the targets of angiogenesis may be a potential antitumor vaccine that could activate the specificity immune response effectively.

Here, we produced a DC-based vaccine via bone marrow generated DCs (BmDCs) pulsed with the recombinant adenovirus encoding full VE-cadherin gene (Ad-VEC), and evaluated its protective and therapeutic effects *in vivo*. We further characterized the chief mechanism of driving its antitumor effect, and elucidated the basic processes necessary for immune-mediated tumor rejection.

## RESULTS

### Culture of mature BmDCs and the expression of VE-cadherin

**Figure 1:**
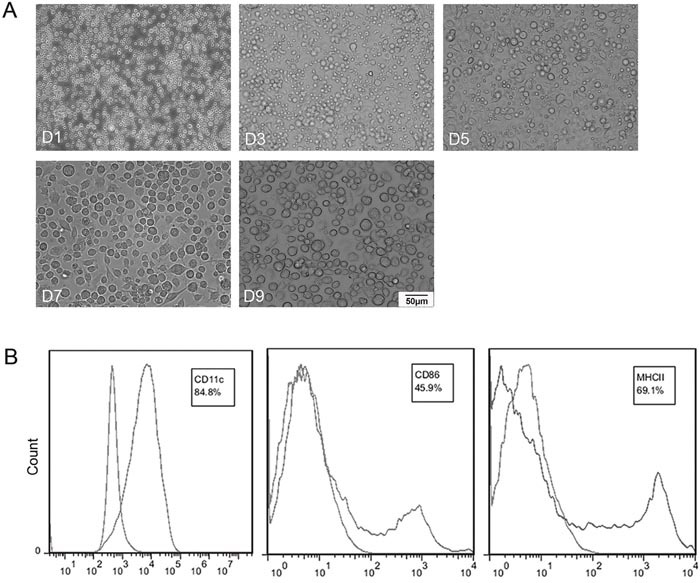
Characterizations of bone marrow generated DCs (BmDCs) **(A)** Hematopoietic progenitor cells from the BALB/c mice were cultured for 7 day and then incubated with Ad-VEC or Ad-Null for another 2 days. D1, D3, D5, D7 and D9 represent the day of cell culture. Mature DCs were observed by optical microscopy (×200). **(B)** Surface phenotype of BmDCs was analyzed by flow cytometry. BmDCs expressed 84.8%, 45.9% and 69.1% of CD11c, CD86 and MHC class II, respectively.

BmDCs were cultured and observed by optical microscope at different time points. Figure 1A shows that mature DCs were large cells with oval, burr or irregularly shaped, and the cellular morphology maintained the same after co-incubation with adenovirus. High expression of DC-associated marker CD11c and the costimulatory molecules CD86 and MHC class II were observed in these cells (Figure 1B). Western blotting showed that transfected DCs expressed VE-cadherin cassette. Thus, we got mature BmDCs targeting VE-cadherin by recombinant adenovirus transfection.

### Induction of protective and therapeutic antitumor immunity

**Figure 2:**
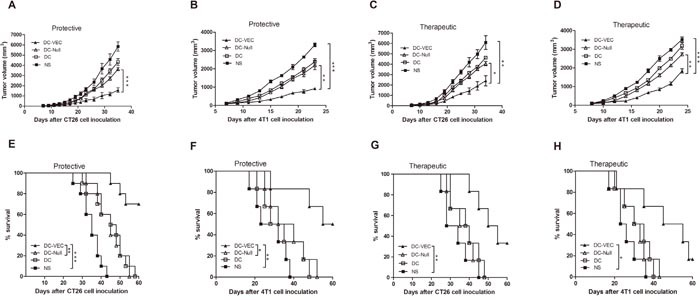
Protective and therapeutic antitumor effect In the protective groups, mice were immunized subcutaneously weekly for three times, then challenged subcutaneously with 5×10^5^ CT26 cells **(A** and **E)** or 4T1 cells **(B** and **F)** seven days after the third immunization. For therapeutic test, mice were treated once a week for four weeks, three days after 5×10^5^ CT26 cells **(C** and **G)** or 4T1 cells **(D** and **H)** were introduced subcutaneously. An apparent decrease of tumor size and survival advantage in DC-VEC group (▲) was seen versus other groups with significant statistical difference. (*p<0.05, **p<0.01, ***p<0.001).

As shown in Figure 2, tumors in mice vaccinated with DC-VEC resulted in retarded tumor progression. Tumor inhibition rates (TIs) are calculated by TI=(V_contral_-V_experiment_)/V_contral_. Compared with NS group, the TIs of DC-VEC group in the protective model were 73.1% for CT26 model at day 35 (Figure 2A), and 71.8% for 4T1 model at day 23 (Figure 2B). In the therapeutic model, the TIs were 60.5% for CT26 model at day 34 (Figure 2C), and 47.8% for 4T1 model at day 24 (Figure 2D). Besides, the overall survival of mice vaccinated with DC-VEC was also significantly longer than that of the control groups in both protective and therapeutic models (Figure 2E-2H). Thus, DC-VEC vaccine shows effective protective and therapeutic antitumor activity *in vivo*.

### Inhibition of angiogenesis

**Figure 3:**
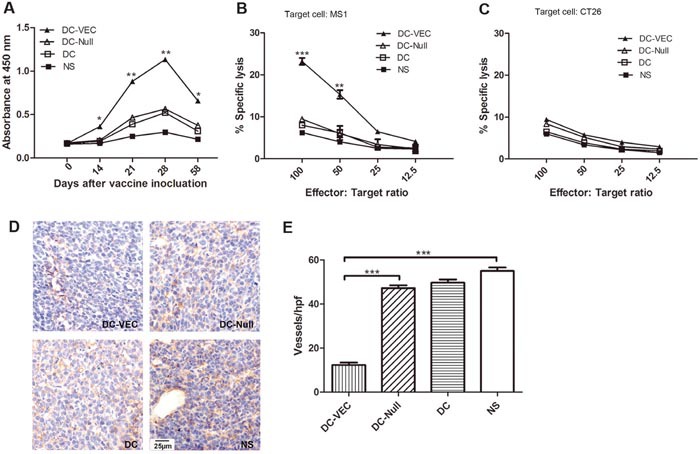
Anti-angiogenesis response *in vivo* **(A)** Analysis of antibody in serum. It shows that significant IgG were generated after the third immunization and the high level of IgG maintained for a long time in DC-VEC group (▲). **(B** and **C)** The killing rates of CTL on different effector/target ratio were calculated. Lymphocytes from DC-VEC group were cytotoxic to MS1 cell **(B)** but not to CT26 cell **(C)**. Stronger CTL response was detected with the graduate increase of effector/target ratios in DC-VEC group, especially at the effector/target ratio of 100:1 and 50:1. **(D)** Immunohistochemical analysis of microvessels in tumor tissues from immunized mice was performed (magnification, ×400). **(E)** Vessel density was determined by counting microvessels in each high-power field (hpf) in the sections. It shows that DC-VEC vaccine resulted in apparent suppression of angiogenesis. All the differences have statistical significance. (*p<0.05, **p<0.01, ***p<0.001).

ELISA was performed to evaluate the generation of vascular endothelial specific antibody by DC-VEC *in vivo*. As shown in Figure 3A, sera immunoglobulin G (IgG) responding to MS1 expressing protein was observed in DC-VEC immunized mice. Though the level of IgG in DC-VEC group was low but detectable after first immunization, and significant antibodies were generated after the third immunization and the high titer of IgG maintained for a long time. In contrast, sera from the control groups (DC-Null, DC, NS) showed rather weaker reactivity.

Then, the CTL assay was performed to quantify the CD8^+^ T lymphocytes activated by DC-VEC vaccine. T lymphocytes isolated from DC-VEC group showed the toxic ability against MS1 cell *in vitro* at dose dependently. At the effector/target ratio of 100:1, T lymphocytes in DC-VEC group exhibited the greatest cytotoxicity (Figure 3B). Nevertheless, no cytotoxicity against CT26 (no VE-cadherin expression) was observed in any group (Figure 3C). It means that DC-VEC vaccine could induce VE-cadherin specific CTL response.

We further performed immunohistochemistry to evaluate the microvessel density in tumor tissue sections. As shown in Figure 3D and 3E, compared with the control groups, DC-VEC vaccine resulted in apparent suppression of angiogenesis, the reduction in vessel density was microscopically examined in the view of high-power field. Altogether, our data demonstrates that DC-VEC vaccine inhibits the tumor-related angiogenesis *in vivo*.

### Induction of immunoregulatory effects and safety

**Figure 4:**
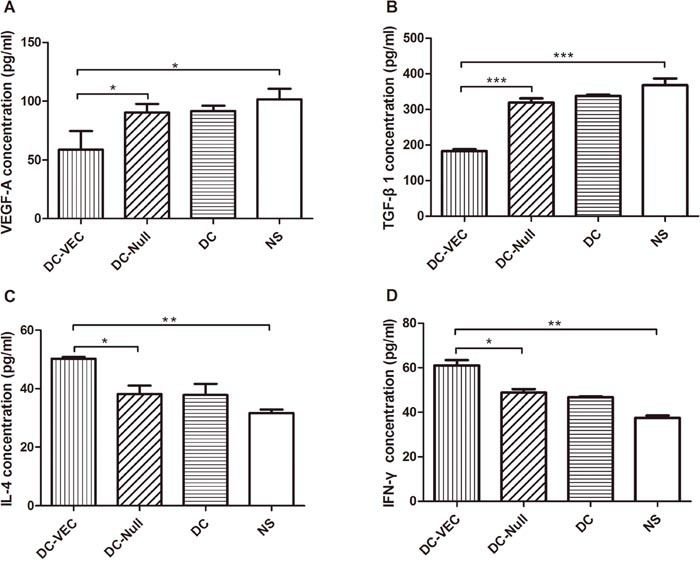
Cytokines analysis by ELISA Mice sera were harvested for the quantitation of **(A)** VEGF-A and **(B)** TGF-β1, and the supernatant of splenocytes was analyzed for **(C)** IL-4 and **(D)** IFN-γ. A significant decrease of VEGF-A and TGF-β1 and an increase of IL-4 and IFN-γ were detected in DC-VEC group (▲). (*p<0.05, **p<0.01, ***p<0.001).

To assess the possible changes of immunoregulation in the anti-tumor activity, ELISAs were conducted to evaluate both mice sera and spleen lymphocytes supernatants. Previous studies demonstrated that VEGF-A and TGF-β were also immune suppressive factors in addition to promoting tumor angiogenesis [22, 23]. IL-4 and INF-γ were active immune cytokines secreted mainly by Th2 and Th1 cells, respectively [24, 25]. As shown in Figure 4, compared with the control groups, the increased production of IL-4 and IFN-γ, and the decreased secretion of VEGF-A and TGF-β1 were seen in the DC-VEC group.

**Figure 5:**
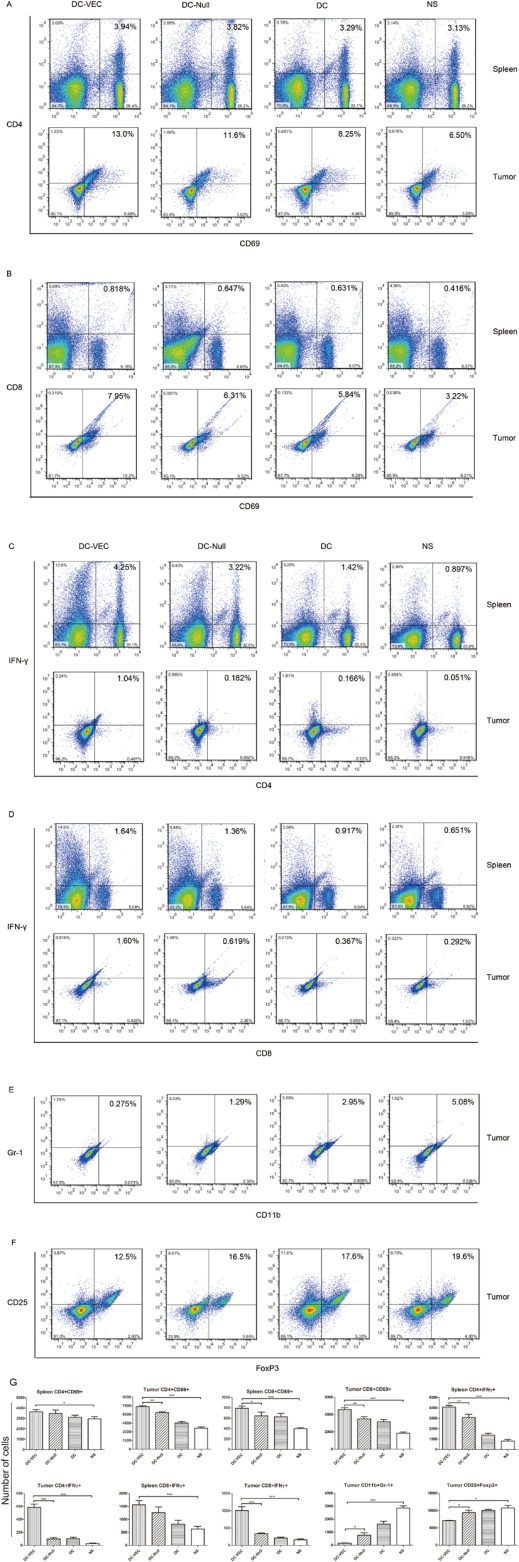
Analyses of activated lymphocytes and IFN-γ by flow cytometry Left panels represent the lymphocytes obtained from DC-VEC group, and the others represent the DC-Null, DC and NS groups, respectively. **(A** and **B)** A trend is seen that activated CD4^+^ and CD8^+^ T cells in DC-VEC group were higher than the controls. **(C** and **D)** T lymphocytes isolated from spleens and tumors in DC-VEC group have increased CD4^+^ IFN-γ^+^ and CD8^+^ IFN-γ^+^ staining. **(E** and **F)** The percentages of CD11b^+^ Gr-1^+^ (MDSCs) and CD25^+^ Foxp3^+^ (Tregs) within the tumor tissues of DC-VEC group are mildly decreased. **(G)** The statistic analyses of the flow cytometry (*p<0.05, **p<0.01, ***p<0.001).

Next, activation of T lymphocytes by vaccination was examined. We analyzed the responsive subsets of T-cell populations and related cytokines in immunized mice. Splenocytes and immune-associated cells in tumors were quantitated by flow cytometry using specific monoclonal antibodies (mAbs). CD4^+^ and CD8^+^ T cells of DC-VEC immunized mice were higher than those of the control groups (Figure 5A and 5B). The IFN-γ secreted by activated CD4^+^ and CD8^+^ T cells was analyzed. Compared with the control groups, the T cells isolated from spleens and tumors of DC-VEC group showed increased CD4^+^ IFN-γ^+^ and CD8^+^ IFN-γ^+^ staining (Figure 5C and 5D). Furthermore, the percentages of CD11b^+^ Gr-1^+^ (myeloid-derived suppressor cells, MDSCs) and CD25^+^ Foxp3^+^ (mainly expressed in regulatory T cells, Tregs) within the tumor tissues of DC-VEC group decreased mildly in DC-VEC group (Figure 5E and 5F).

**Figure 6:**
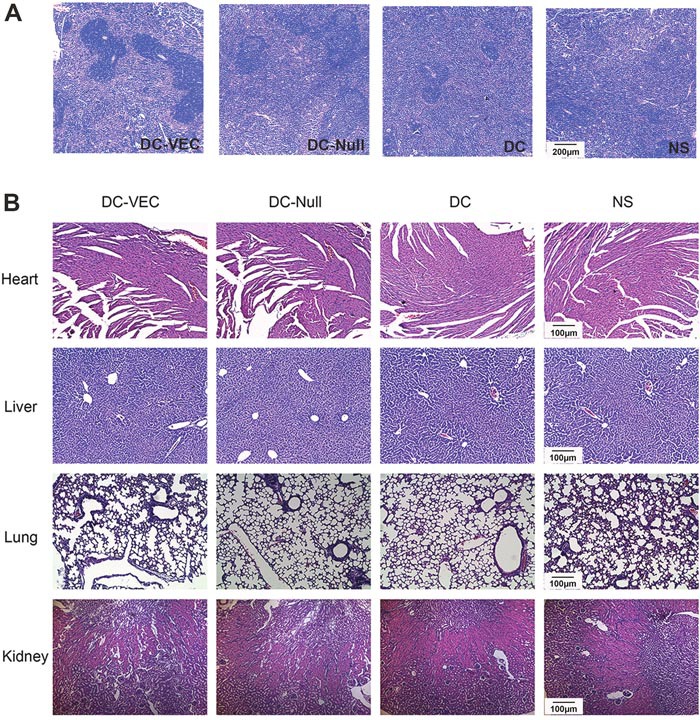
Formation of spleen germinal centers and safety evaluation H&E staining of organs (spleen, heart, liver, lung and kidney) from immunized and tumor challenged mice was conducted. It shows that **(A)** mice in DC-VEC group had more germinal centers in spleen than the controls (magnification, ×50), and **(B)** there are no pathologic changes of major organs in all the groups (magnification, ×100).

Finally, H&E staining of spleen in DC-VEC group generated more germinal centers than the controls (Figure 6A). Also, H&E staining of major organs including heart, kidney, and lung showed that mice treated with DC-VEC had no obvious pathologic changes, such as necrosis, edema, hemorrhage, etc. (Figure 6B). Collectively, these data suggest that DC-VEC vaccine is succeed in modulating the immunoregulation effect *in vivo* and rather safe.

## DISCUSSION

Here we report a strategy for tumor immunotherapy by DC-based anti-angiogenesis. VE-cadherin modified DC vaccine (DC-VEC) shows robust protective and therapeutic antitumor immunity *in vivo*. The main mechanism of antitumor effect is associated with its anti-angiogenesis and immunoregulation response.

Currently, the selective targeting of DC-specific endocytic receptors by linking the relevant antigens or ligands was the most widely studied approach to activate T lymphocytes. To our knowledge, suitable targets may include antigens that cannot be down-regulated by tumor, particularly those directly implicated in malignant behavior, to minimize the chances of immune escape. Because monovalent specificity against a single antigenic peptide is unlikely to work well in patients with a large tumor burden and the heterogeneity of tumor cells, it is possible that vaccination against multiple tumor-associated antigens expressing on endothelial or stromal cells in tumor might be efficient, with the development of multiple tumor antigens being explored.

As we all know, tumor growth is widely dependent on the angiogenesis including proliferation of endothelial cells which are genetically stable. Accumulating evidence has shown that tumor vasculature, characterized by immature vessels, irregular blood flow, tortuous architecture, and VEGF-induced hyperpermeability, is uniquely different from normal vasculature [26–28], thus we speculate that tumor neovascularization may be more vulnerable than normal wound healing. Besides, large amount of vascular endothelial growth factor (VEGF) is secreted by tumor cells to promote angiogenesis during tumorigenesis process. The molecules of VE-cadherin are assembled in the adherens junction during the normal physiological conditions, but in the presence of VEGF, VEGFR2 associates and phosphorylate VE-cadherin, leading to the disruption of the adherens junction. Thus, the level of VE-cadherin phosphorylation is increased significantly in tumors [29, 30]. And compatible with previous reports [12, 17, 31], there are no pathological changes to heart, liver, lung or kidney in our study, demonstrating that VE-cadherin antagonists may act as effective anti-tumor drugs without affecting normal blood vessels. It had been showed that vaccination with soluble fetal liver kinase-1 (flk1) protein pulsed DCs or flk-1/IFN-γ fusion gene transfected DCs could effectively inhibit tumor angiogenesis and metastasis in mice. A strong CTL response destroying endothelial cells was detected and flk1-specific neutralizing antibody was present in immunized mice [32, 33]. Besides that, VE-cadherin represents a typical endothelial cell-specific cadherin. The restricted distribution and unique biological function distinguish it as an attractive target for anti-angiogenesis.

Our study shows that DC-VEC vaccine was succeed in inducing endothelial cell specific humoral immunity. For one thing, cytokines secreted by activated T lymphocytes through the antigen presenting process could stimulate B cells involved in the humoral immunity [34]. Moreover, several findings suggest that DCs may directly activate B cell maturation [6, 7, 35]. For another, CD8^+^ CTLs are important effector arm in antitumor immunity. The CTL activity is mostly mediated by CD8^+^ T cells and MHC class I restricted [36]. In our study, VE-cadherin specific CTL response was tested to investigate the priming of CD8^+^ T cells-mediated response after prophylactic vaccination with DC-VEC *in vivo*. The effector cells obtained from spleen of DC-VEC group could lyse the MS1 cell with VE-cadherin expression, while not the VE-cadherin negative parallel CT26 cell. The immunohistochemistry demonstrated that DC-VEC vaccine resulted in the reduction in vessel density. In a word, DC-VEC vaccine could inhibit the tumor angiogenesis *in vivo*.

Besides, it can be excluded that DC-VEC vaccine participated in immunoregulation effectively in our study. By immunochemistry and ELISA, we found that the spleen and tumor tissues in DC-VEC group had increased number of activated CD4^+^ and CD8^+^ T cells and cytotoxicity cytokines of IFN-γ and IL-4, with decreased secretion of immune suppressive cells and cytokines. At the same time, more germinal centers were generated in spleen of DC-VEC group, which means that when the body is stimulated by antigens, a portion of activated B cells may enter into the primary lymphoid follicles from thymus dependent area of the peripheral immune organs, forming germinal center by cellular differentiation and proliferation. Antigen-special immune response induced by B cells occurs mainly in the germinal center once again [37].

One critical step in the malignant progression of incipient tumors is evasion and suppression the host immune system. The prevalent mechanism of immune evasion is via the suppressive activity of MDSCs, Tregs, etc. [23, 38]. MDSCs are mobilized during tumorigenesis and infiltrate developing tumors by promoting tumor vascularization and disrupting major mechanisms of immunosurveillance [39]. Tregs have diverse immune modulatory functions in cancer. Similar to MDSCs, Tregs suppress tumor-associated antigen presentation and interfere with cytotoxic T cell function by inhibiting cytolytic granule release [40]. Additionally, quite a few studies reported that endothelial cells are associated with immune-suppression, and tumor cells can amplify the immune-suppression effect by inducing restrictive endothelial cells [22, 41]. In our study, DC-VEC vaccine could re-educate the immunosuppressive activity of MDSCs and Tregs, and decrease the secretion of immunosuppressive factors VEGF-A and TGF-β. It may be resulted from the anti-angiogenesis effect induced by DC-VEC vaccine, which is against the immune-suppression effect caused by endothelial cells. Certainly, its specific mechanism needs to be determined. Besides, there have been several targeted drugs available against tumor angiogenesis so far, such as Bevacizumab (Avastin^®^), which has been widely used for the treatment of metastatic colorectal cancer. These anti-angiogenesis drugs need to be administrated repeatedly to take the optimal anti-tumor effect. DC vaccines are probably of key importance to initiate immune response and permit establishment of immunological memory. Therefore, DC-VEC vaccine is a potential antitumor vaccine that could activate the specificity immune response effectively. Compared with anti-angiogenesis therapy merely, DC-VEC vaccine could avoid the tumor immune escape and conquer the limitation of repeatedly continuous dosing, inducing robust antitumor immunity.

In conclusion, our findings provide proof-of-concept that vaccination with DC-VEC could be a promising approach for cancer immunotherapy via its effects of anti-angiogenesis and immunoregulation. Our work may contribute to the design of novel vaccine for tumor immunotherapy.

## MATERIALS AND METHODS

### Mice and cell lines

Female (6–8 weeks old) BALB/c mice were purchased from Laboratory Animal Center of Beijing HFK Bioscience. Mice were housed in a specific pathogen-free environment and treated in accordance with the guidelines established by the Animal Care and Use Committee of the Sichuan University, China. The murine colon carcinoma cell line CT26, the murine breast carcinoma 4T1, the normal murine endothelial cell line MS1 and the human embryonic kidney cell line HEK293 were purchased from ATCC (American Type Culture Collection, VA, USA). These cells were routinely maintained in DMEM supplemented with 10% fetal calf serum plus 1% ampicillin.

### Generation and characteristics of BmDCs

BmDCs were generated as previously described with some modifications [42]. Briefly, bone marrow was collected from tibias and femurs of female BALB/c mice, passed through a nylon mesh to remove small pieces of bone and debris, then resuspended in RPMI 1640 medium containing 10% fetal bovine serum, GM-CSF (10 ng/ml), IL-4 (10 ng/ml) and 1% ampicillin, cultured in tissue culture dishes for five days. Then immature DCs were further incubated and stimulated with lipopolysaccharide (LPS) (100 ng/ml) also with GM-CSF (10 ng/ml), IL-4 (10 ng/ml) for two days to induce maturation. Mature DCs were observed by Olympus optical microscope.

Expression analysis of surface molecules was quantitated by flow cytometry using the following mAbs against MHC class II (PE-conjugated), CD11c (APC-conjugated) and CD86 (FITC-conjugated) (all obtained from Biolegend). Species and isotype-matched mAbs were used as controls. For flow cytometry, aliquots of 1×10^6^ BmDCs were incubated with the mAbs for 40 min at 4°C. The cells were washed with PBS twice and subsequently analyzed by flow cytometer (NovoCyte).

### Construction of VE-cadherin modified DC vaccines

Generation, purification and characterization of recombinant adenovirus (Ad-VEC, Ad-Null) have been described earlier [17]. In short, total RNA was extracted from mouse fetal tissue and cDNA encoding the entire VE-cad sequence was amplified by RT-PCR. The sense primer was 5’ ata gtc gac cga agg atg cag agg ctc aca 3’ with a Sal I linker at the 5’ end; The antisense primer was 5’ gcg aag ctt acc cta gat gat gag ttc ctc ctg 3’ with a Hind III linker and a termination codon at the 5’ end. Recombinant adenovirus (Ad-VEC) was generated by inserting the amplified sequence into the multiple cloning site of pShuttle plasmid. The Ad-Null virus was a control, which was identical to Ad-VEC except for lacking the gene of interest. The recombinant adenovirus was amplified in HEK293 cells. For gene introduction, mature BmDCs were infected with Ad-VEC or Ad-Null for two days at 37°C. The nonadherent cells were then collected and were mainly DCs (referred to as VE-cadherin modified DCs). At this time point, in order to ascertain the optimal quantity of Ad-VEC infecting DCs, expression of the recombinant cassette was confirmed by western blotting of transfected DCs lysates. Vaccine protein was probed with mouse anti-VE-cadherin mAb followed by horseradish peroxidase (HRP)-conjugated secondary anti-mouse antibody.

### Immunization protocol and yumor challenge

Before immunization, Ad-VEC, Ad-Null pulsed or unpulsed 9-day-cultured BmDCs (DC-VEC, DC-Null, DC) were collected and washed three times with PBS. A total of 1×10^6^ DCs in 0.1 ml PBS were injected subcutaneously (s.c.) in the lower left flank of syngeneic BALB/c mice. To investigate the protective effect of DC-VEC in tumor models, mice were randomly divided into four groups and injected s.c with DC-VEC, DC-Null, DC or NS (normal saline 0.9% NaCl). Each mouse received three immunizations every other week. Seven days after the third immunization, mice were injected s.c. in the right flank with 5×10^5^ tumor cells (CT26 or 4T1). All mice were bled 5-7 days after each immunization and sera were stored at −20°C.

For therapeutic effect analysis, mice were injected with 5×10^5^ live tumor cells s.c. in the right flank for tumors challenge. When the tumors were visible and palpable, mice were treated with injection of the vaccines s.c. weekly for four weeks. All experiments were performed by using individual groups of 10 mice. Tumor volume (mm^3^) was estimated by tumor width and length, which was measured every 2-3 days by calipers [43]. Mice were monitored for the onset of tumor growth (∼1mm^3^) and sacrificed for humane reasons when tumors grew to 20 to 25 mm (longest diameter).

### ELISAs

ELISAs were performed for detecting sera antibodies and cytokines in each group. Briefly, 96-well plates coated with MS1 cells were incubated with diluted mice sera, and tagged using HRP-conjugated anti-mouse IgG. In addition to analyze cytokine secretion, mice spleen cells were harvested and cultured five weeks after tumor challenge. Supernatants from T cell cultures were analyzed for the presence of cytokines IL-4 and IFN-γ by ELISA using commercially available kits (Dakewe Biotech). Sera from mice seven days after the last immunization were harvested for the quantitation of cytokines VEGF-A and TGF-β1. Assays were performed in triplicate.

### Flow cytometry of splenocytes and tumor-infiltrated cells

Mice immunized and challenged with CT26 cells were sacrificed five weeks after tumor inoculation. Splenocytes were harvested and cultured overnight at 37°C, then were used for staining. Cells within the tumor microenvironment were isolated and harvested from mice two weeks after immunization and tumor challenge by 0.1% collagenase. Antibodies used to phenotype the cells were anti-CD4-APC, anti-CD8-FITC, anti-CD69-PE, anti-IFN-γ-PE, anti-CD11b-APC, anti-Gr-1-FITC, anti-CD25-PE and anti-FoxP3-FITC (Biolegend).

### Cytotoxic T lymphocyte (CTL) assay

Lactate dehydrogenase (LDH) cytotoxicity assay kit (GenMed Scientifics) was performed to detect possible VE-cadherin specific cytotoxicity mediated by CTLs. Splenocytes were harvested and pooled from mice seven days after the last immunization using commercial lymphocyte separation medium (Dakewe Biotech). Splenocytes were incubated with target cells MS1 or CT26 at different effector/target ratios for 4 h at 37°C. The specific lysis activity was calculated by the formula: cytotoxicity=(experimental release−spontaneous release)/(maximum release−spontaneous release)×100%. Spontaneous release is the standard negative control (in absence of effector cells), and maximum release represents 100% LDH release by lysis of target cells.

### Immunohistochemistry and H&E staining

Mice were sacrificed five weeks after tumor inoculation. The paraffin-embedded tissues were cut into 6-μm-thick sections for staining. H&E staining was performed to evaluate the safety of the vaccine and identify the germinal centers in spleen. Immunohistochemistry staining was used to evaluate the microvessels density. Sections were probed with a murine endothelial antibody against CD34 (Abcam), then incubated with biotinylated secondary antibody following with streptavidin-biotin complex at 37°C for 40 min, respectively (ZSGB-Bio). The numbers of CD34-positive microvessels were microscopically examined in the high-power field of view. These slides were observed by Olympus optical microscope.

### Statistics

Data are presented as Mean ± SD (standard deviation). Differences between groups were tested by performing ANOVA, Student's t-test and Kaplan-Meier analysis. All the p values were two-sided and p<0.05 was considered to indicate statistical significance (*p<0.05, **p<0.01, ***p<0.001).

## Abbreviations

DC: dendritic cell
APC: antigen-presenting cell
CTL: cytotoxic T lymphocyte
VE-cadherin: vascular endothelial-cadherin
BmDC: bone marrow generated DC
TI: tumor inhibition rate
IgG: immunoglobulin G
IL-4: interleukin-4
IFN-γ: interferon-gamma
VEGF-A: vascular endothelial growth factor A
TGF-β1: transforming growth factor beta-1
Tregs: regulatory T cells
MDSCs: myeloid-derived suppressor cells

## References

[1] Meixlsperger S, Leung CS, Ramer PC, Pack M, Vanoaica LD, Breton G, Pascolo S, Salazar AM, Dzionek A, Schmitz J, Steinman RM, Munz C (2013). CD141+ dendritic cells produce prominent amounts of IFN-alpha after dsRNA recognition and can be targeted via DEC-205 in humanized mice. Blood.

[2] Sabado RL, Meseck M, Bhardwaj N (2016). Dendritic Cell Vaccines. Vaccine Design: Methods Mol Biol..

[3] Schreibelt G, Bol KF, Westdorp H, Wimmers F, Aarntzen EH, Duiveman-de Boer T, van de Rakt MW, Scharenborg NM, de Boer AJ, Pots JM (2016). Effective clinical responses in metastatic melanoma patients after vaccination with primary myeloid dendritic cells. Clin Cancer Res.

[4] Banchereau J, Steinman RM (1998). Dendritic cells and the control of immunity. Nature.

[5] Hart DN (1997). Dendritic cells: unique leukocyte populations which control the primary immune response. Blood.

[6] Shin C, Han JA, Koh H, Choi B, Cho Y, Jeong H, Ra JS, Sung PS, Shin EC, Ryu S (2015). CD8α-Dendritic Cells Induce Antigen-Specific T Follicular Helper Cells Generating Efficient Humoral Immune Responses. Cell reports.

[7] Sornasse T, Flamand V, De Becker G, Bazin H, Tielemans F, Thielemans K, Urbain J, Leo O, Moser M (1992). Antigen-pulsed dendritic cells can efficiently induce an antibody response in vivo. The Journal of experimental medicine.

[8] Kastenmuller W, Kastenmuller K, Kurts C, Seder RA (2014). Dendritic cell-targeted vaccines--hope or hype?. Nature reviews Immunology.

[9] Figdor CG, de Vries IJ, Lesterhuis WJ, Melief CJ (2004). Dendritic cell immunotherapy: mapping the way. Nature medicine.

[10] Anguille S, Smits EL, Lion E, van Tendeloo VF, Berneman ZN (2014). Clinical use of dendritic cells for cancer therapy. The lancet oncology.

[11] Vacchelli E, Vitale I, Eggermont A, Fridman WH, Fučíková J, Cremer I, Galon J, Tartour E, Zitvogel L, Kroemer G (2013). Trial watch: Dendritic cell-based interventions for cancer therapy. Oncoimmunology.

[12] Liao F, Doody JF, Overholser J, Finnerty B, Bassi R, Wu Y, Dejana E, Kussie P, Bohlen P, Hicklin DJ (2002). Selective targeting of angiogenic tumor vasculature by vascular endothelial-cadherin antibody inhibits tumor growth without affecting vascular permeability. Cancer research.

[13] Coon BG, Baeyens N, Han J, Budatha M, Ross TD, Fang JS, Yun S, Thomas JL, Schwartz MA (2015). Intramembrane binding of VE-cadherin to VEGFR2 and VEGFR3 assembles the endothelial mechanosensory complex. The Journal of cell biology.

[14] Yamamoto H, Ehling M, Kato K, Kanai K, van Lessen M, Frye M, Zeuschner D, Nakayama M, Vestweber D, Adams RH (2015). Integrin β1 controls VE-cadherin localization and blood vessel stability. Nature communications.

[15] Barry AK, Wang N, Leckband DE (2015). Local VE-cadherin mechanotransduction triggers long-ranged remodeling of endothelial monolayers. J Cell Sci.

[16] Corada M, Liao F, Lindgren M, Lampugnani MG, Breviario F, Frank R, Muller WA, Hicklin DJ, Bohlen P, Dejana E (2001). Monoclonal antibodies directed to different regions of vascular endothelial cadherin extracellular domain affect adhesion and clustering of the protein and modulate endothelial permeability. Blood.

[17] Zhao Z, Yao Y, Ding Z, Chen X, Xie K, Luo Y, Zhang J, Chen X, Wu X, Xu J (2011). Antitumour immunity mediated by mannan-modified adenovirus vectors expressing VE-cadherin. Vaccine.

[18] Zhao Y, Adjei AA (2015). Targeting angiogenesis in cancer therapy: moving beyond vascular endothelial growth factor. The oncologist.

[19] Zhang M, Ye G, Li J, Wang Y (2015). Recent advance in molecular angiogenesis in glioblastoma: the challenge and hope for anti-angiogenic therapy. Brain tumor pathology.

[20] Khan K, Cunningham D, Chau I (2015). Targeting angiogenic pathways in colorectal cancer: complexities, challenges and future directions. Curr Drug Targets.

[21] Wagner SC, Ichim TE, Ma H, Szymanski J, Perez JA, Lopez J, Bogin V, Patel AN, Marincola FM, Kesari S (2015). Cancer anti-angiogenesis vaccines: Is the tumor vasculature antigenically unique?. Journal of translational medicine.

[22] Mulligan JK, Young MR (2010). Tumors induce the formation of suppressor endothelial cells in vivo. Cancer immunology, immunotherapy.

[23] Quail DF, Joyce JA (2013). Microenvironmental regulation of tumor progression and metastasis. Nature medicine.

[24] Maggi E, Parronchi P, Manetti R, Simonelli C, Piccinni M, Rugiu FS, De Carli M, Ricci M, Romagnani S (1992). Reciprocal regulatory effects of IFN-gamma and IL-4 on the in vitro development of human Th1 and Th2 clones. The Journal of Immunology.

[25] Nurieva RI, Chung Y (2010). Understanding the development and function of T follicular helper cells. Cellular & molecular immunology.

[26] Roberts WG, Palade GE (1997). Neovasculature induced by vascular endothelial growth factor is fenestrated. Cancer Res.

[27] Benjamin LE, Golijanin D, Itin A, Pode D, Keshet E (1999). Selective ablation of immature blood vessels in established human tumors follows vascular endothelial growth factor withdrawal. The Journal of clinical investigation.

[28] Lanitis E, Irving M, Coukos G (2015). Targeting the tumor vasculature to enhance T cell activity. Current opinion in immunology.

[29] Wallez Y, Vilgrain I, Huber P (2006). Angiogenesis: the VE-cadherin switch. Trends in Cardiovascular Medicine.

[30] Cavallaro U, Liebner S, Dejana E (2006). Endothelial cadherins and tumor angiogenesis. Exp Cell Res.

[31] Corada M, Zanetta L, Orsenigo F, Breviario F, Lampugnani MG, Bernasconi S, Liao F, Hicklin DJ, Bohlen P, Dejana E (2002). A monoclonal antibody to vascular endothelial cadherin inhibits tumor angiogenesis without side effects on endothelial permeability. Blood.

[32] Li Y, Wang MN, Li H, King KD, Bassi R, Sun H, Santiago A, Hooper AT, Bohlen P, Hicklin DJ (2002). Active immunization against the vascular endothelial growth factor receptor flk1 inhibits tumor angiogenesis and metastasis. J Exp Med.

[33] Pan J, Heiser A, Marget M, Steinmann J, Kabelitz D (2005). Enhanced antimetastatic effect of fetal liver kinase 1 extracellular domain and interferon-gamma fusion gene-modified dendritic cell vaccination. Gene therapy.

[34] Reuschenbach M, von Knebel Doeberitz M, Wentzensen N (2009). A systematic review of humoral immune responses against tumor antigens. Cancer immunology, immunotherapy.

[35] Dubois B, Vanbervliet B, Fayette J, Massacrier C, Van Kooten C, Briere F, Banchereau J, Caux C (1997). Dendritic cells enhance growth and differentiation of CD40-activated B lymphocytes. J Exp Med.

[36] Schoenberger SP, Toes RE, van der Voort EI, Offringa R, Melief CJ (1998). T-cell help for cytotoxic T lymphocytes is mediated by CD40–CD40L interactions. Nature.

[37] Victora GD, Nussenzweig MC (2012). Germinal centers. Annual review of immunology.

[38] Lindau D, Gielen P, Kroesen M, Wesseling P, Adema GJ (2013). The immunosuppressive tumour network: myeloid-derived suppressor cells, regulatory T cells and natural killer T cells. Immunology.

[39] Talmadge JE, Gabrilovich DI (2013). History of myeloid-derived suppressor cells. Nature reviews Cancer.

[40] von Boehmer H, Daniel C (2013). Therapeutic opportunities for manipulating T(Reg) cells in autoimmunity and cancer. Nature reviews Drug discovery.

[41] Ziogas AC, Gavalas NG, Tsiatas M, Tsitsilonis O, Politi E, Terpos E, Rodolakis A, Vlahos G, Thomakos N, Haidopoulos D (2012). VEGF directly suppresses activation of T cells from ovarian cancer patients and healthy individuals via VEGF receptor type 2. International Journal of Cancer.

[42] Inaba K, Inaba M, Romani N, Aya H, Deguchi M, Ikehara S, Muramatsu S, Steinman R (1992). Generation of large numbers of dendritic cells from mouse bone marrow cultures supplemented with granulocyte/macrophage colony-stimulating factor. The Journal of experimental medicine.

[43] Sauter BV, Martinet O, Zhang WJ, Mandeli J, Woo SL (2000). Adenovirus-mediated gene transfer of endostatin in vivo results in high level of transgene expression and inhibition of tumor growth and metastases. Proceedings of the National Academy of Sciences.

